# Evidence of Crimean-Congo Haemorrhagic Fever Virus Occurrence in Ixodidae Ticks of Armenia

**Published:** 2019-03-30

**Authors:** Hasmik Gevorgyan, Gohar G. Grigoryan, Hripsime A. Atoyan, Martin Rukhkyan, Astghik Hakobyan, Hovakim Zakaryan, Sargis A. Aghayan

**Affiliations:** 1 Scientific Center of Zoology and Hydroecology, Yerevan, Armenia; 2 National Institute of Health, MOH RA, Yerevan, Armenia; 3 Institute of Molecular Biology NAS RA, Yerevan, Armenia; 4 Laboratory of Zoology, Research Institute of Biology, Yerevan State University, Yerevan, Armenia

**Keywords:** CCHF, Ixodid ticks, Armenia, ELISA, Infection rate

## Abstract

**Background:**

Crimean-Congo hemorrhagic fever (CCHF) causes serious health problems in humans. Though ticks of the genera *Hyalomma* play a significant role in the CCHF virus transmission it was also found in 31 other tick species.

**Methods:**

Totally, 1412 ticks from 8 remote sites in Armenia during 2016 were sampled, pooled (3–5 ticks per pool) and tested for the presence of CCHFV antigen using ELISA test.

**Results:**

From 359 tick pools, 132 were CCHF virus antigen-positive. From 6 tick species, four species (*Rhipicephalus sanguineus*, *R. annulatus*, *R. bursa*, *Hyalomma marginatum*) were positive for the virus antigen and *R. sanguineus* was the most prevalent (37.9%). *Dermacentor marginatus* and *Ixodes ricinus* revealed no positive pools, but both revealed delectable but very low virus antigen titers. The highest infection rate (50%) was observed in *R. sanguineus*, whereas *H. marginatus* rate of infection was 1 out of 17 pools.

**Conclusion:**

For the first time in the last four decades CCHF virus antigen was detected in Ixodid ticks of Armenia. This finding substantiates the role of *R. sanguineus* in the disease epidemiology; however, the role of *H. marginatum* in the CCHF virus circulation in the country could not be excluded.

## Introduction

Ixodid ticks present significant epidemiologic and health concerns in the transmission of dangerous arboviruses to humans (1, 2). In addition, the potential for person to person transmission makes the control of the spread of infection more difficult and unpredictable (3, 4). The Crimean-Congo hemorrhagic fever (CCHF) is one of the zoonotic viral infections that manifests with influenza-like symptoms and can progress to hemorrhagic disease with mortality rate ranging from 5% to 50% (5, 6). There are several routes and sources of human infection with CCHF virus, e.g., a direct tick bite, contact with virus-containing killed ticks, direct contact with blood and other tissues of infected animals or humans (3, 7–9). The causative agent is CCHF virus (CCHFV), a member of the Bunyaviridae, genus Nairovirus. The virus has been isolated from at least 31 species of ticks belonging to *Ixodida*e (hard ticks) and *Argasidae* (soft ticks) families (10).

A number of ticks in genera like *Rhipicephalus* and *Dermacentor* are able to care CCHF virus, but ticks of the genus *Hyalomma* are the essential vector for the pathogen transmission (11, 12). Except direct, through bites, disease transmission there are additional factors that can potentially increase the virus survival in nature. The livestock maintains the stable circulation of the virus in nature. The virus replicates to high titers in some organs of the carrier livestock and usually causes only subclinical disease in domestic animals, that goes unnoticed and untreated (13). The member of the crow family (Corvidae) are the reservoir of the ticks immature stage, whereas, infected animals are the main reservoirs for fully developed adult stage of ticks (14).

Epidemics and sporadic CCHF cases have been reported from Eastern Europe, Russia, Africa, the Middle East, and Central Asia (15–17).

Tick-born arbovirus infections present epidemiologic and health challenge in Armenia, as well. Being represented by a distinct variety of landscape zones with several climate zones in relatively small territory, the countryside supports potential distribution of the vector tick species (18). Although historical review related to arbovirus surveillance in Armenia has revealed the circulation of different arthropod-transmitted viruses (19), the first detection of CCHFV in ticks and the only laboratory-confirmed severe case of human CCHFV disease in Armenia is dated back to 1970s (20). To the best of our knowledge there are no published reports about CCHFV activity in the country in the last 5 decades.

In spite of recent worsening of CCHFV situation in neighboring Turkey and Iran (9, 21–24), there is no consistent monitoring of CCHF key vectors distribution and abundance in Armenia. Moreover, there are no efforts to determine the prevalence and the level of CCHFV in ixodid ticks.

This study was designed to study the possible presence of CCHF virus in ixodid ticks in target provinces of Armenia. The initial studies are highly relevant in the context of transformed socio-economic conditions, climatic and environmental changes, and particularly in the context of a significant interest towards tick-born arboviral infections worldwide (25).

## Materials and Methods

**Fig. 1.**
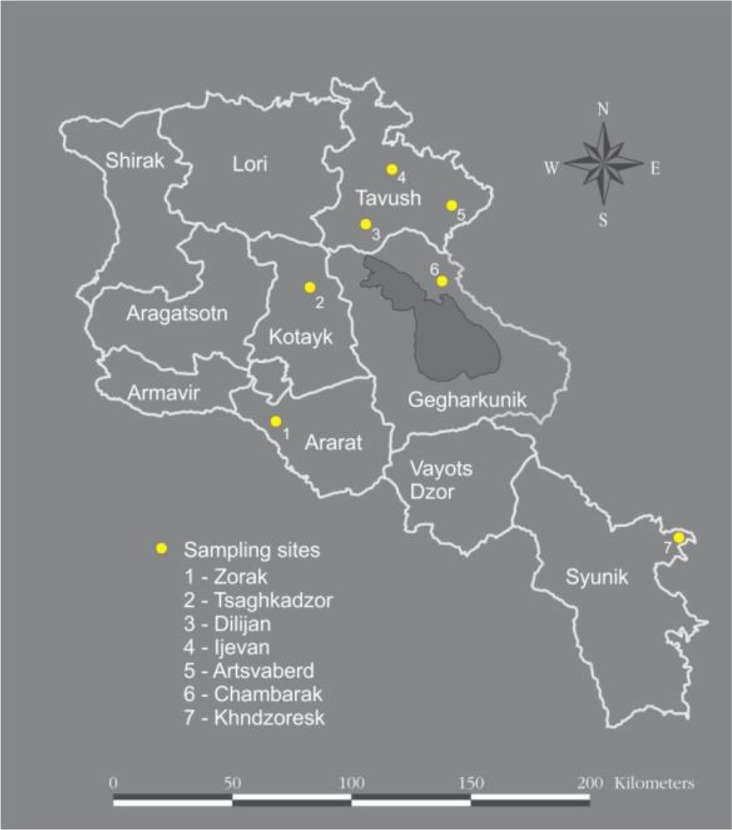
Study area

The ticks’ collection was conducted in 2016 and covered 8 locations in five provinces of Armenia that included southern, central and northern parts of the country (Fig. 1). The selection of these territories was based on preliminary but limited knowledge of high level of *Hyalomma* and *Rhipicephalus* ticks abundance in these areas (26). Ticks were collected using two methodologies - by flagging in pasture lands of each locality and direct collection of the ticks from livestock.

The sampling was linked to the ticks’ seasonal activities and covered period from Apr to Oct 2016 (27). The field sampling of free-living questing ticks was performed using flannel strips (1000×100mm). At each pastureland, 5 areas were randomly selected. Each selected area was walked in a slow manner dragging the flannel through the grass. At the end of each area caught ticks were collected. For collection of ticks from host animals (cattle and sheep), 10 animals were randomly selected for examination in each herd. Ticks were collected with forceps. Each individually collected tick was placed in separate vials, labeled and kept alive until species identification. Species identification was implemented under the light microscope with magnification of 100×, using the published taxonomic keys and species accounts (28).

Overall, 1412 ticks were sampled of which only 548 were identified to species level. In 864 cases the identification was difficult due to inadequate life stage or the damage of the sample. However, these “unidentified species” were pooled in groups according to sampling area and tested separately to determine whether or not these ticks may contain any titer of CCHFV antigen.

Prior to serological detection of CCHFV antigen, each collected tick was rinsed with 70% ethanol and then placed in physiological solution with antibiotics (mixture of 100U/mL penicillin, 100μg/mL streptomycin). Sampled ticks were clustered and labeled according to genera, collection area, season, and placed in separate vials and stored at −18 °C for further processing.

Stored ticks were pooled into groups of three to five (depending on size) by species and collection area. All pooled ticks were put in the liquid nitrogen for 20min then homogenized using glass pestle. Homogenized samples were resuspended in 200μl of dilution buffer in case of flat ticks and 400μl in case of engorged ticks. After the short vortexing tubes were centrifuged at 2000rpm for 5min and supernatant was collected to test for CCHF viral antigen using antigen-capture ELISA (VectoCrimea-CHF ELISA, Vector-Best, Russia) (29). The sensitivity and specificity of the standard panels of positive and negative samples were indicated as 100%. Based on obtained optical density (OD) values we calculated critical OD (ODcr) according to the following formula: ODcr= ODm (K−)+ 0.2 where ODm (K−) is the medium value of negative controls’ OD. The sample was considered as positive if OD sample was higher than ODcr. According to the manufacturer protocol if the OD sample was higher than 0.8*ODcr but less than ODcr the reading was considered as inconclusive, i.e., doubtful. The data were analyzed using previously published technique developed for pooled sample studies (30).

## Results

Of 1412 collected ticks only 548 were identified to species level which belonged to four genera of the family *Ixodidae*: *Rhipicephalus*, *Hyalomma*, *Ixodes*, *Dermacentor.* The dominant tick species were from the genera *Rhipicephalus* (364 ticks, 66.4%). Further, 6 tick species were identified, and *R. sanguineus* was identified as the most common tick species (n= 208, 37.9%) followed by *R. annulatus* (n= 68, 12.4%), *R. bursa* (n= 88, 16%), *D. marginatus* (n= 64, 11.7%), *I. ricinus* (n= 53, 9.5%) and *H. marginatum* (n= 67, 12.4%).

The analysis of tick species according to geographic location showed that *Rhipicephalus* spp. was the most abundant tick species in Kotayk region. *I. ricinus* and *R. annulatus* are the most recorded species in Gegharkunik Province, while sheep in Ararat and Tavush provinces suffered from *D. marginatus* ticks.

Collected ticks were separated into 137 pools of identified tick species and 222 pools of not identified and assayed using ELISA test. From 137 pools comprising six tick species, 38 tested positive for the CCHFV antigen. These pools included four tick species (*R. sanguineus*, *R. annulatus*, *R. bursa*, *H. marginatum*) and were registered in all surveyed areas. Noteworthy, *R. sanguineus* showed the highest CCHFV infection rate meanwhile only 1 of 17 *H. marginatum* pools showed positive response. The other six species contained doubtful (non-interpretable) pools (Table 1).

**Table 1.** Serological results of Crimean-Congo hemorrhagic fever antigen detection in pools according to tick species*

**Tick species**	**No ticks**	**Infection rate**	**No Pools**	**Positive pools**	**Doubtful pools**
***Rhipicephalus annulatus***	65	107.73	17	6	1
***Dermacentor marginatus***	68	0.00	16	0	2
***Hyalomma marginatum***	70	17.85	17	1	3
***Ixodes ricinus***	51	0.00	13	0	1
***Rhipicephalus bursa***	86	71.87	22	5	3
***Rhipicephalus sanguineus***	208	175.30	52	26	4
**Total**	**548**		**137**	**38**	**14**

* Infection rate per 1000 ticks and 95% confidence interval

Identified and non-identified tick pools were also clustered to analyze occurrence of CCHF virus antigen in carriers in targeted geographic areas. Totally, 359 pools were tested. The observation of ticks’ infection rate in studied areas is shown in Table 2.

**Table 2.** Results of CCHFV antigen detection in tick pools according to geographic location*

**Town/village (Province)**	**Infection rate**	**No Pools**	**Positive Pools**	**Doubtful Pools**
**Zorak (Ararat)**	0.00	16	0	2
**Chambarak (Gegharqunik)**	42.80	39	6	2
**Tsaghkadzor (Kotayk)**	195.79	37	20	3
**Khndzoresk (Syunik)**	10.87	25	1	2
**Dilijan (Tavush)**	156.65	220	100	18
**Ijevan (Tavush)**	65.76	8	2	0
**Artsvaberd (Tavush)**	73.58	14	3	3
**Total**		**359**	**132**	**30**

* Infection rate per 1000 ticks and 95% Confidence Interval

The highest tick infection level was found in Kotayk and Tavush Provinces. Ticks from other areas demonstrated uneven distribution, with lower prevalence in southern and central part of the country.

## Discussion

Changes in climatic, environmental, social and anthropogenic factors have contributed to the spread of CCHF infection in new areas and increased incidence in endemic regions. Armenia is one of the countries where no studies on circulation of CCHF virus among ticks and livestock were conducted during last 5 decades. The first alarming data on the CCHF risk in Armenia occurred after the detection of AGDP antibodies to the virus in cattle sera from 5 areas of the country (31). The next evidence of the virus was the isolation of CCHF strains from *H. marginatum* (5), *H. anatolicum* (1), *R. bursa* (1), *Boophilus* (*Rhipicephalus*) *annulatus* (1), *R. rossicus* (1) (32). We have embarked on this study based on these 2 references (31, 32). The specific study areas were selected based on preliminary known distribution of *Hyalomma* and *Rhipicephalus* ticks in certain territories, as well as contrasting bio-climatic conditions between targeted provinces.

Our samples were represented predominately by *Rhipicephalus*, *Hyalomma*, *Ixodes*, *Dermacentor* genera distinguished by their high magnitude of populations and variety of domestic and wild animal hosts (15, 18). Ticks were identified, pooled and analyzed using ELISA according to species and geographic location. From 1412 collected tick samples by the time ticks were delivered to the laboratory the condition of 60% of ticks were not appropriate for the species identification. Since the aim of this study was to determine the presence of CCHF virus in ticks in a specific area identified for the study, we pooled together the unidentified ticks and analyzed these pools only according to geographic area.

The *Rhipicephalus* is the most prevalent genus in Kotayk region. *I. ricinus* and *R. annulatus* are the most prevalent species in Gegharkunik Province. The most abundant tick species in the current study is *R. sanguineus* which is, in general, in agreement with published data (33, 34).

Comparison of results obtained from different regions of the county showed that ticks collected from central regions were more infected than those from southern and northern regions. First, this can be explained by the composition of sampled ticks from this region, where *R. sanguineus* the most abundant species that were also showing the highest infection rate among all other tick species. Second, it could be due to the concentration of livestock and the quality of breeding management including poor hygienic conditions of livestock breeding sites.

Among all areas studied, we identified the highest ticks’ infection rate in Kotayk Province with prevalence of 54.05%. The second highest prevalence was identified in Dilijan town (Tavush Province) where along with *R. sanguineus* two more species (*I. ricinus* and *R. bursa*) were obtained. Prevalence of infected ticks was decreased in the north – Ijevan (25%) and Artsvaberd (21.5%) as well as in the east -Chambarak (15%). According to our data in Artsvaberd and Ijevan the most abundant tick species were *R. bursa* and in Chambarak - *R. annulatus*, demonstrating that the infection prevalence in study sites coincides with tick species abundance and infection rates. While in previously published study no virus was observed in *H. detritum*, *H. scupense*, *I*. *ricinus*, or *Argas persicus* (31), in our study one pool of *I. ricinus* and 2 pools of *D. marginatum* demonstrated low-level titers of CCHFV antigen. Some of ticks could be the virus carriers and these finding may suggest that further studies may be necessary to determine the role of these other tick species in the epidemiology of the CCHFV transmission. In our opinion, a significant attention should be devoted to the recognized vector of CCHF virus - *H. marginatum*. In our study from 17 *H. marginatum* ticks only one pool showed positive response and two were rated as doubtful. If compared with *Rhipicephalus* genera figures, there are indications to consider the latter as an acceptable vector for the virus in Armenia. At the same time, however, relatively high number of unidentified tick species with significant number of positive pools (132) may avert from an objective assessment of *Hyalomma* impact on CCHF cases in Armenia. Therefore, a geographically wider area and more directed studies should be conducted to be able to answer this question.

According to scientific consensus, although, many tick genera are capable of becoming infected with CCHF virus, several species of genus *Hyalomma* are the principal vector for CCHF virus. To some extent our results and the only serologically proven human CCHF case recorded in 1974 (20) support this statement. *Hyalomma* and *Rhipicephalus* ticks are widely distributed and abundant in Armenia, but *Rhipicephalus* spp. is likely to have a greater role in the circulation of CCHFV in Armenia and by serving as a virus reservoir. Hence, the occurrence of the pathogen in ticks can represent a significant risk for human population and this risk should not be overlooked.

Further studies should be focused on animals’ seroprevalence and virus genetic diversity for identification of high-risk areas for human infection.

## Conclusion

For the first time in the last five decades, CCHF virus antigen was detected in tick samples in Armenia. Serological test (ELISA) enabled to prove the existence of CCHFV antigen mostly in *Rhipicephalus* ticks. Our results showed the important role of *R. sanguineus* tick species in supporting of CCHFV circulation in the natural foci, although without officially registered cases of human CCHF. Moreover, the highest level of infection based on collection area is registered in Kotayk region which coincided with abundance of *R. sanguineus*. Our results demonstrated the necessity to conduct PCR-based studies to determine genetic diversity of CCHFV in the country. Further, country-wide investigations including surveys of domestic animal sera and the risk assessment of human exposure to infected tick bite may be necessary. This data can be used as foundation for development of a country-wide epidemiologic study to identify unrecognized CCHF foci in Armenia.
